# A Cross-Sectional Survey on the Use of Tobacco, Attempts on Cessation, and Locus of Control Among College Students

**DOI:** 10.7759/cureus.46195

**Published:** 2023-09-29

**Authors:** Sachin Shinde, Sonali Kumari, Manish Prakash, Prashant G M, Anmol Khajuria, Nitin Modi, Gaurav Kumar Chhabra, Priyanka Paul

**Affiliations:** 1 Department of Orthodontics and Dentofacial Orthopedics, Prakash Institute of Medical Sciences, Islampur, IND; 2 Department of Periodontology, Vananchal Dental College and Hospital, Garhwa, IND; 3 Department of Oral Medicine and Radiology, Vananchal Dental College and Hospital, Garhwa, IND; 4 Department of Public Health Dentistry, College of Dental Sciences and Hospital, Davanagere, IND; 5 Department of Public Health Dentistry, MM College of Dental Sciences and Research, Mullana, IND; 6 Department of Prosthodontics, Vyas Dental College and Hospital, Jodhpur, IND; 7 Department of Public Health Dentistry, NIMS Dental College and Hospital, Wardha, IND; 8 Department of Public Health Dentistry, Sharad Pawar Dental College and Hospital, Datta Meghe Institute of Higher Education And Research, Deemed to be University, Wardha, IND

**Keywords:** external locus, internal locus, locus of control, tobacco cessation, tobacco use

## Abstract

Background

Socio-cultural considerations (such as drug availability) and psychological traits play a significant role in predicting whether a person will use drugs in the future and dependency on the drugs. Second-, third-, and fourth-hand smoking and E-cigarettes are influencing factors for the use of tobacco in college students. This study conducted research to ascertain whether there is a potential relationship between tobacco consumption and various factors, including internal and external control sites and demographics.

Materials and methods

Participants in the study were found by walk-up distribution at multiple campus-wide smoking places, department announcements, and on-campus advertisements. Social media and participant references were also used in this study as recruitment tools. In addition, the locus of control questionnaire also identifies if the participating individual had extrinsic or intrinsic reinforcing routines. The classification of the participating individuals into respective internal and external locus of control was in accordance with their response survey after which a statistical analysis was done.

Results

This study found an association between smoking on campus and reported attempts to quit. Additionally, there is a strong association (r(85) = 0.31, p < 0.01) between reported tobacco use status and cigarette use on campus. Participants' gender and smoking status also had r(85) = 0.39, p-value < 0.01 correlation. The bulk of respondents indicated that they were seniors and off-campus living concluding for 36% (n = 34) and 60% (n = 51) of the total. Twenty-seven percent (n = 24) of the respondents were first-year college students and the rest 33% (n = 29) said their parents had no college education at all or incomplete college education.

Conclusion

Whenever there is a strong perception of organizational support for anti-tobacco policies, and improving compliance, there is a drastic increase in cigarette cessation and a drop in tobacco usage among those who still smoke. Perceived organizational support is strongly and positively connected with cessation among the organization's members.

## Introduction

Smoking and smokeless tobacco use have increased among young and adolescent students [1]. College students are at the most risk for the initiation of tobacco and alcohol use. Cigarette smoking is a trend among young and adult students, usually studying in high school and college. Tobacco use can be in the form of smoke and smokeless tobacco, depending on the availability of its resources in specific areas. Cigarette smoking is a prevalent cause of morbidity and mortality in India [2,3]. More than one million deaths annually in India are due to tobacco use. The preventive efforts should be practical, so it is crucial to comprehend the variables that affect the consumption and abuse of alcohol and tobacco. In addition to sociocultural considerations (such as drug availability), psychological traits play a significant role in predicting whether a person will use drugs in the future [4-6]. Second, third, and fourth-hand smoking and E-cigarettes influence tobacco use in college students.

There has been limited research on substance misuse, although locus of control (LoC) has been studied in detail in the psychological and social scientific literature. The LoC is the degree to which people believe they have control over the outcome of events in their lives, as opposed to external forces (beyond their influence). Julian B. Rotter developed the concept in 1954, and it has since become an aspect of personality psychology. Constructs associated with LoC, like self-efficacy, have been evaluated in specific studies of cigarette and alcohol users [7-9]. There is evidence that LoC is linked to substance use/abuse. However, there is limited information about the link between LoC orientation and young adults' use of alcohol and smoking as compared to adult behaviors. Younger adults with substance abuse have been the subject of more extensive research, like quitting and abstinence [10].

This study identifies whether there was an association between tobacco consumption and factors like internal, and external control and demographic details. Qualitative data analysis was conducted to classify participating individuals by their expectations and behavior concerning demographic factors such as age, gender, and habits.

## Materials and methods

Participants and procedure

The study participants comprised male and female smokers enrolled in undergraduate and graduate programs at a university.

Study design and setting

This present cross-sectional study was done at Dental College and Hospital, Jaipur, Rajasthan, India.

Study participants

Participants in the study were found by walk-up distribution at multiple campus-wide smoking places, department announcements, and on-campus advertisements. Social media and participant references were also used in this study as recruitment tools. Everyone who uses tobacco, including those thinking about quitting, has tried to stop or has tried and failed to do so, was eligible for the study. Smokers, chewers, cigars, cigarillos, and everyone else who uses tobacco in any way were all considered tobacco users. After being contacted, tobacco users on campus were told to visit the website listed on the handout and complete the survey.

Institutional Review Board (IRB)

After obtaining ethical approval from the Institutional Ethics Committee (NIMSUR/IEC/2023/112), NIMS Dental College and Hospital, NIMS University, Jaipur, India. The participants had signed an informed consent form which indicated that they had given their voluntary consent to be a part of the research.

Questionnaire development

The survey was created using questions from the joint Global Adult Tobacco Survey of the Centres for Disease Control and the World Health Organization (C.D.C. & WHO, 2010). The questionnaire, which identifies whether participating individuals had extrinsic or intrinsic reinforcing routines, was also included in the study. The participating individuals were classified into an internal locus of control (ILoC) and external locus of control (ELoC) (IV) as per their responses. After the survey, the researchers collected the data, and statistical analysis was done. Participants were requested to differentiate self-inventory to see if there was a relationship between certain demographic factors, LoC, and tobacco consumption based on subgroups identified from survey data.

Sample size estimation

The sample size is determined using the following formula

 n = z 1-α/2 2 p(1-p)

 d2

where,

p = previous expected values=0.27

d =desired Margin of error = 0.5

Z1-α/22 confidence interval of 95%, z = 1.96

sample size: 100

Statistical analyses

This research study survey was used to find an association between tobacco consumption and factors like ILoC and ELoC and demographics. The survey findings were qualitatively analyzed to group respondents by behavioral potential and expectations about demographic factors like age and gender. The variations in the means between groups were calculated using frequency analysis and correlations after the participant's mean LoC scores were computed using SPSS (IBM Corp. Armonk, NY). The level of Significance was set at p value of less than 5% (p ≤ 0.05).

## Results

The survey was conducted and 100 responses to the survey were received out of 150. Fifteen of those respondents were eliminated because they refused to give informed consent. Data collected from 85 students were evaluated in this study (43 male, 42 female). There is a significant correlation observed between tobacco usage and habits, which proves the positive relation of tobacco use on college campuses as seen in Tables 1, 2.

**Table 1:** Co-relation of the tobacco usage with students’ habits. **Correlation is significant at the 0.01 level (two-tailed)

Survey variables			
Variable	Pearson Correlation	P-value	N
What is the status of your tobacco use?	0.379**	0.004	85
Do you smoke in campus?	0.311**	0.004	85

**Table 2:** Co-relation of gender and tobacco usage. **Correlation is significant at the 0.01 level (two-tailed)

Correlated survey variables			
Variable	Pearson Correlation	P-value	N
gender	0.314**	0.001**	85
How much do you use tobacco?	0.326**	0.001**	85

The mean for the ILoC subscale was reported to be 30, while the standard for the ELoC subscale was noted to be 19. There is a correlation of gender with tobacco usage in which maximum tobacco use was observed in males than females. A highly statistically significant difference was observed in the use of tobacco among males and females in Table 3.

**Table 3:** Co-relation of tobacco use and quit attempts. *Correlation is significant at the 0.05 level (two-tailed) There is a significant correlation between tobacco usage and quit attempts. Students with high tobacco usage have tried maximum quit attempts.

Survey variables			
Variable	Pearson Correlation	P-value	N
Have you attempted to quit?	0.210*	0.04	85
Do you use tobacco on campus?	0.210*	0.04	85

The results suggest that the study's college smokers have a higher ILoC, which supports the research premise. It is vital to remember that LoC is a continuous evaluation and can change depending on a person's psychological state and various factors relating to their health. The “powerful others” subscale received an average score of roughly 10 from the participants. The internal and powerful scale variable has shown a high significant variable scale correlation. The powerful others subscale measures the opinion of a person that perceived gatekeepers like doctors, nurses, other healthcare workers, administrators, and policymakers have an impact on their health. Most respondents, 78% (n = 69), were young adults between 18 and 24.

Most respondents included seniors 36% (n = 34) and 60% (n = 51) students living off-campus. Of the respondents, 27% (n = 24) identified themselves as first-year college students, and 33% (n = 29) said their parents had no college education.

No association was observed between tobacco users, attempts to quit, family earnings, or the most significant level of education found when the LoC correlation was estimated. But r(85) = 0.24 at p < 0.05. This study found a link between smoking on campus and reported attempts to quit. Additionally, there is a strong association (r(85) = 0.31, p < 0.01) between noted tobacco use and cigarette consumption on campus. Participants' gender and smoking status also correlated (p<0.01), r(85) = 0.39 seen in Table 4.

**Table 4:** Internal and powerful scale variable correlations. *Correlation is significant at the 0.05 level (two-tailed) **Correlation is significant at the 0.01 level (two-tailed)

LoC variables	1	2	3	4	5	6	7	8	9	10	11 12
1. Willpower to recover from my illness	-										
2. I'm in charge of my own health	0.05	-									
3. My fault for being ill	0.04	1.01**	-								
4. Self-care is essential to my wellbeing	0.03	1.01**	1.02**	-							
5. I'm sick because I don't look after myself	0.21*	0.32**	0.41**	0.52**	-						
6. I can maintain my health if I take care of myself	0.32**	0.53**	0.42**	0.62**	0.54**	-					
7. Doctor visits reduce the likelihood of being ill	0.40**	0.152	0.10	0.15	0.23*	0.19	-				
8. I can only keep my health up by getting professional advice	0.04	1.01**	1.02**	1.02**	0.24*	0.19	0.16	-			
9. Other people play a big part in whether I’m healthy or sick	0.04	1.01**	1.02**	1.03**	0.22	0.27**	0.16	1.00**	-		
10. Medical professionals maintain my health.	0.29	0.05	0.01	0.14	0.52**	0.13**	0.10	0.16	0.06	-	
11. My recovery is dependent on the care I receive from other people	0.04	1.11**	1.10**	1.10**	0.41**	0.12**	0.16	1.00**	1.00*	0.06	-
12. I maintain my health by heeding medical advice	0.14	0.45**	0.21	0.56**	0.51**	0.55**	0.07	0.31**	0.42*	0.29**	0.30**

Together the ILoC and ELoC and the scale of the other that are powerful showed substantial connections, mainly when it came to the idea that influential people have a significant impact on recovery and health as well as personal authority for one's health. These variables had a p-value < 0.01 with a cheerful correlation seen in Table 5.

**Table 5:** Correlation of External Scale locus of control with questionnaire. *Correlation is significant at the 0.05 level (two-tailed) **Correlation is significant at the 0.01 level (two-tailed)

LoC variables	1	2	3	4	5	6	7	8	9	10	11 12
1. Whatever I do, I'm going to get sick											
2. It seems like random events have a big impact on my health	0.41**										
3. When I am sick I just have to let nature run its course	-0.10	-0.30									
4. When I remain healthy, I am fortunate	-0.10	-0.30	1.10**								
5. Even when I take care of myself it is easy to get sick	0.32**	0.36*	0.34**	0.41**							
6. The fact that I get sick is fate	0.13**	0.42**	0.44**	0.30**	0.51**						
7. Regularly visiting a good doctor will reduce my likelihood of developing health issues	0.38*	0.38*	0.26	0.26	0.12**	0.23					
8. I can only maintain my health by consulting professionals	-0.10	-0.30	1.10**	1.10**	0.41**	0.22*	0.14				
9. I depend a lot on other people to stay healthy	-0.11	0.13	1.10**	1.10**	0.33**	0.50**	0.16	1.00**			
10. Health professionals keep me healthy	0.19*	0.46**	0.07	0.06	0.39**	0.52**	0.20	0.06	0.06		
11. The kind of care I get will determine how quickly I recover	0.15	-0.25	1.10**	1.10**	0.51**	0.28**	0.16	1.10**	1.00**	0.06	
12. The best way for me to stay healthy is to adhere to medical advice	0.04	0.22	0.23	0.32	0.23*	0.46**	0.17	0.41**	0.22*	0.26**	-0.20**

The people may hold intrinsic and extrinsic reinforcement beliefs and reveal ratings on both scales displays substantial relations between the 12 ILoC and ELoC. There is a strong correlation between Internal and External Scale LoC as seen in Table 6.

**Table 6:** Correlations between Internal and External Scale locus of control. *Correlation is significant at the 0.05 level (two-tailed) **Correlation is significant at the 0.01 level (two-tailed)

LoC Variables	1	2	3	4	5	6	7	8	9	10	11 12
1. Willpower to recover from my illness	-										
2. I'm in charge of my own health	0.03	-									
3. My fault for being ill	0.04	1.10**	-								
4. Self-care is essential to my wellbeing	0.05	1.00**	1.00**	-							
5. I'm sick because I don't look after myself	0.23*	0.27**	0.32**	0.47**.	-						
6. I can maintain my health if I take care of myself	0.33**	0.59**	0.41**	0.67**	0.56**	-					
7. Doctor visits reduce the likelihood of being ill	0.34**	-0.20	-0.20	-0.20	0.34**	0.13	-				
8. I can only keep my health up by getting professional advice	0.43**	-0.20	-0.20	-0.20	0.28**	0.16	0.51**	-			
9. Other people play a big part in whether I’m healthy or sick	0.05	1.00**	1.00**	1.00**	0.37**	0.53**	-0.20	0.20	-		
10. Medical professionals maintain my health	0.05	1.00**	1.00**	1.00**	0.36**	0.22	-0.20	-0.20	1.00* *	-	
11. My recovery is dependent on the care I receive from other people	0.11	0.25*	0.32*	0.16	0.35**	0.31*	0.34**	0.26*	0.35**	0.51**	-
12. I maintain my health by heeding medical advice	0.13	0.20**	0.20**	0.27**	0.31**	0.37**	0.23**	0.41**	0.42**	0.50**	0.54** -

## Discussion

This study found that college students have strong ILoC. It has been confirmed by previous studies showing that tobacco users in both academic settings and workplaces are predominantly internal [11-16]. Healthcare professionals should consider the consistency of smokers in numerous studies, including the one above, to design successful interventions to affect patients. Indeed, some might argue that LoC is inaccurate for existing tobacco interventions. For instance, several health behavior therapies entail informing the patient about the 14 potential benefits and hazards of quitting.

ILoC representatives may not react as positively to courses, leaflets, and commercials as ELoC representatives. In-house employees prefer self-directed, practical applications instead of educational materials [17,18]. Research suggests that components such as the one obtained in the Transtheoretical Model (T.T.M.) and the Health Belief Model (HBM) benefit ILoC patients. However, these tools may be more relevant to specific health characteristics. It was developed to raise awareness and education. Research suggests that components such as those found in the Transtheoretical Model (T.T.M.) and the HBM might be more beneficial to ILoC patients. Still, these tools benefit specific health awareness and education aspects more. Hypertheoretical modeling fits this paradigm of effective ILoC treatment, moving from pre-contemplation to contemplation, planning, action, and ultimately management. This line is consistent with Rotter's SLT framework. It is because its structure requires treatment to propel the patient through each stage according to their expectations and enhancement values. For example, a person would only consider changing his behavior after first understanding the benefits of subsequent reinforcement. However, the action phase of TTM and its maintenance phase would ultimately be caused by the person's expectation to change successfully.

Policy changes or other motivations may generate preparedness [19]. ILoC patients may be more likely to be affected by their expectations due to how much importance they place on their individual preferences and capacities. Therefore, low anticipation could severely harm an ILoC patient's chances of successfully changing behavior. Marks (1998) further claims that social factors strongly impact practitioners when creating and implementing therapies for 15 ELoC. It may cause practitioners to classify their patients as external mistakenly. The idea that people having ELoC have increased chances of developing stress, which may promote to use of tobacco, may lead practitioners to reflexively design therapies that focus on the ELoC [20].

However, Rotter's SLT reinforcement value construct makes it unlikely that these interventions will be successful because, irrespective of assumptions or beliefs, a person with an ILoC does not appreciate or pursue the same reinforcement as a counterpart with ELoC [21]. The perceived failures of cigarette interventions may be significantly impacted when patients are misdiagnosed of having ELoC and later given a suboptimal intervention (the once-matching LoC to intervention), mainly while pupils are classified as primarily internal. Another intriguing aspect of LoC among college students was highlighted by the research that the students were more on ILoC (31) than external [22].

Also, the student got 10 points. It is a relatively low rating given the severity of her ILoC measured. However, other solid scale ratings do not represent this difference in LoC. Policy and environmental issues are thought to significantly impact patients with internal LoC, which others have evaluated very strongly [23]. This is logical, as managers and employers often formulate regulations where students smoke [16]. “Policies to limit smoking behavior in the workplace by minimizing smoking opportunities, reducing incentives to smoke, and improving social support for quitting,” argues Moskowitz.

Additionally, they looked at the effect of a smoking ban at work. They discovered that 26.4% of smokers in cities with rigorous smoking ban policies reported quitting within six months of the study and were still leaving at follow-up, compared to only 19.1% in areas where no ban has been implemented. The researchers discovered a link between the effectiveness of anti-smoking regulations and the influence of such rules on smoking behavior [18].

It is crucial to note, though, the researchers also claimed that these findings could only be reached in areas where residents were aware of anti-tobacco legislation and regulations. The research conducted in 1999 by Farkas et al. [19], and Goyal et al. [24] examines how policies and social acceptability affect smoking. The authors found that when there is a strong perception of organizational support for anti-tobacco policies, there was a rise in quitting cigarettes and also a drop in tobacco consumption among those participants who were still smoking as well as that perceived organizational support which is strongly and positively connected with cessation among the organization's members. According to research [20,24], smoking bans at home can also help people quit smoking by limiting their access to tobacco. It creates a significant impact in working with patients with ILoC. As reported by Ahamed et al., an innovative way of tobacco cessation, like tobacco cessation by prescription, should be tried to see the success rates of tobacco cessation [25]. Furthermore, people who wanted to stop smoking for a week or 17 more days are likelier than those who failed to quit within the next 18 months.

Limitation

As the sample size of the population is small leading to limited access to the information due to less sample size and confined geographic location.

## Conclusions

The LoC concept gauges a person's belief in their capacity to influence their circumstances based on their actions. People who have an ILoC perceive their actions to have an impact on their experiences. People who have an ELoC believe that their fate is out of their hands and is instead decided by luck, fate, chance, or strong others. ILoC and ELoC have sown a massive impact on health and health-related problems so more studies should be conducted to promote better health outcomes among different age groups.

## References

[1] Lassi G, Taylor AE, Mahedy L, Heron J, Eisen T, Munafò MR (2019). Locus of control is associated with tobacco and alcohol consumption in young adults of the Avon Longitudinal Study of Parents and Children. R Soc Open Sci.

[2] Sheffer C, MacKillop J, McGeary J (2012). Discounting, locus of control, and cognitive impulsiveness independently predict tobacco dependence treatment outcomes in a highly dependent, lower socioeconomic group of smokers. Am J Addict.

[3] Mokdad AH, Marks JS, Stroup DF, Gerberding JL (2004). Actual causes of death in the United States, 2000. JAMA.

[4] Munafò M, Johnstone E, Murphy M, Walton R (2001). New directions in the genetic mechanisms underlying nicotine addiction. Addict Biol.

[5] Munafò MR, Black S (2007). Personality and smoking status: a longitudinal analysis. Nicotine Tob Res.

[6] Hakulinen C, Hintsanen M, Munafò MR, Virtanen M, Kivimäki M, Batty GD, Jokela M (2015). Personality and smoking: individual-participant meta-analysis of nine cohort studies. Addiction.

[7] Shope JT, Copeland LA, Maharg R, Dielman TE, Butchart AT (1993). Assessment of adolescent refusal skills in an alcohol misuse prevention study. Health Educ Q.

[8] Karimy M, Niknami S, Heidarnia AR, Hajizadeh E, Shamsi M (2013). Refusal self efficacy, self esteem, smoking refusal skills and water pipe (Hookah) smoking among iranian male adolescents. Asian Pac J Cancer Prev.

[9] Wills TA, Gibbons FX, Sargent JD, Gerrard M, Lee HR, Dal Cin S (2010). Good self-control moderates the effect of mass media on adolescent tobacco and alcohol use: tests with studies of children and adolescents. Health Psychol.

[10] Heidari M, Ghodusi M (2016). Relationship of assess self-esteem and locus of control with quality of life during treatment stages in patients referring to drug addiction rehabilitation centers. Mater Sociomed.

[11] Zadarko E, Zadarko BP, Barabasz Z (2010). Tobacco smoking prevalence among students from Euro region Eastern Carpathians. Przegl Lek.

[12] Schmitz N, Neumann W, Oppermann R (2000). Stress, burnout and locus of control in German nurses. Int J Nurs Stud.

[13] Ahamed MS, Chhabra KG, Reche A, Paul P (2022). Tobacco cessation by prescription - a 180 degree turn. J Family Med Prim Care.

[14] Sabbagh HJ, Abdelaziz W, Quritum M (2022). Cigarettes' use and capabilities-opportunities-motivation-for-behavior model: a multi-country survey of adolescents and young adults. Front Public Health.

[15] El Tantawi M, Sabbagh HJ, Alkhateeb NA (2022). Oral manifestations in young adults infected with COVID-19 and impact of smoking: a multi-country cross-sectional study. PeerJ.

[16] Quadri MF, Lusher J, Folayan MO (2023). Factors associated with an increase in tobacco use and alcohol drinking during the COVID-19 pandemic: A cross-sectional study of data from 105 countries. Tob Induc Dis.

[17] Sheffer C, Mackillop J, McGeary J (2012). Delay discounting, locus of control, and cognitive impulsiveness independently predict tobacco dependence treatment outcomes in a highly dependent, lower socioeconomic group of smokers. Am J Addict.

[18] Peltzer K (2001). Tobacco use among black South African university students: attitudes, risk awareness and health locus of control. Curationis.

[19] Partlak Günüşen N, Ustün B, Erdem S (2014). Work stress and emotional exhaustion in nurses: the mediating role of internal locus of control. Res Theory Nurs Pract.

[20] Prochaska JO, DiClemente CC (1983). Stages and processes of self-change of smoking: toward an integrative model of change. J Consult Clin Psychol.

[21] Rotter JB (1966). Generalized expectancies for internal versus external control of reinforcement. Psychol Monogr.

[22] Moskowitz JM, Lin Z, Hudes ES (2000). The impact of workplace smoking ordinances in California on smoking cessation. Am J Public Health.

[23] Farkas AJ, Gilpin EA, White MM, Pierce JP (2000). Association between household and workplace smoking restrictions and adolescent smoking. JAMA.

[24] Goyal A, Sharma A, Agarwal S, Bhansali S, Chhabra KG, Chhabra C (2020). Determinants of tobacco use among children of a rural village in India: an exploratory qualitative study. Asian Pac J Cancer Prev
..

[25] Riad A, Al-Khanati NM, Issa J, Zenati M, Abdesslem NB, Attia S, Krsek M (2022). Oral health-related knowledge, attitudes and behaviours of Arab dental students: multi-national cross-sectional study and literature analysis 2000-2020. Int J Environ Res Public Health.

